# Variation of karyotype and nuclear DNA content among four species of *Plectranthus* L’ Héritier, 1788 (Lamiaceae) from Brazil

**DOI:** 10.3897/CompCytogen.v9i4.6255

**Published:** 2015-09-08

**Authors:** Thaís Furtado Nani, Amanda Teixeira Mesquita, Fernanda de Oliveira Bustamante, Sandro Barbosa, João Vítor Calvelli Barbosa, Lisete Chamma Davide

**Affiliations:** 1 Laboratory of Cytogenetics, Biology Department, Federal University of Lavras, Lavras-MG, PO Box 3037, Brazil; 2 Laboratory of Biosystematics and Pollination, Plant Biology Department, University of Campinas, Campinas- SP, Monteiro Lobato Street, 255, Cidade Universitária Zeferino Vaz Barão Geraldo, Zip Code 13083-862, Brazil; 3 Laboratory of Environmental Biotechnology & Genotoxicity, Institute of Natural Sciences, Federal University of Alfenas, Alfenas-MG, Gabriel Monteiro da Silva Street, 700, Zip Code 37130-000, Brazil

**Keywords:** Cytogenetics, Cytotaxonomy, Flow cytometry, Karyotypic evolution

## Abstract

*Plectranthus* is a genus which includes species of ornamental and medicinal potential. It faces taxonomic problems due to aggregating species previously belonging to the genus *Coleus*, a fact that has contributed to the existence of various synonymies. The species *Plectranthus amboinicus*, *Plectranthus barbatus*, *Plectranthus grandis* and *Plectranthus neochilus* are included in this context. Some authors consider *Plectranthus barbatus* and *Plectranthus grandis* as synonyms. The present work was carried out with the aim of comparing plants of the above-mentioned species, originating from different localities in Brazil, with regards to chromosome number and karyotypic morphology, correlated to the nuclear DNA content. There was no variation in chromosome number among plants of the same species. *Plectranthus amboinicus* was the only species to exhibit 2n=34, whereas the others had 2n=30. No karyotypic differences were found among the plants of each species, except for *Plectranthus barbatus*. The plants of the *Plectranthus* species revealed little coincidence between chromosome pairs. The nuclear DNA content allowed grouping *Plectranthus amboinicus* and *Plectranthus neochilus*, with the highest mean values, and *Plectranthus grandis* and *Plectranthus barbatus* with the lowest ones. Differences in DNA amount among the plants were identified only for *Plectranthus barbatus*. These results allow the inference that the populations of *Plectranthus amboinicus* and *Plectranthus neochilus* present coincident karyotypes among their plants, and *Plectranthus grandis* is probably a synonym of *Plectranthus barbatus*.

## Introduction

The family Lamiaceae Martinov, 1820 contains approximately 250 genera and 6,500 species (Anon 2014). Its main area of distribution is the Mediterranean region and Mid-East, up to Central Asia (Chengyih and Hsiwen 1982). In Brazilian territory, 500 species of 34 genera can be found broadly distributed across the country (Harley et al. 2013).

*Plectranthus* L’ Héritier, 1788 is one of the most common genera of this family, and comprises about 300 species (Richardson 1992) that generally serve medicinal and ornamental interest (Thoppil 1993). Species of this genus are native to tropical and subtropical regions of Africa, Australia, the East Indies, the Malay Archipelago, and the Philipines (Lebowitz 1985).

This genus, along with *Burnatastrum* Briquet, 1897, *Coleus* Loureiro, 1790 *Englerastrum* Briquet, 1894, *Isodictyophorus* Briquet, 1917 and *Neomullera* unrecorded, has already been placed in the genus *Ocimum* Linnaeus, 1753. *Coleus* and *Plectranthus* have been considered distinct genera only because of morphological differences of the stamen; however, this characteristic has later been considered insufficient for the separation of these taxa. This way, the *Coleus* species were aggregated to the genus *Plectranthus*, turning this grouping into a unique genus and independent of *Ocimum* Linnaeus, 1753 (Morton 1962). The taxonomic history of *Plectranthus* has contributed to some of its species being known by many different synonymies (Lukhoba et al. 2006).

In Brazil there are some important species of this genus used as herbal medicines. *Plectranthus amboinicus* (Loureiro, 1825) Sprengel, 1825 is native to East Asia, later introduced in Cuba and distributed in America (Castillo and Gonzalez 1999). *Plectranthus barbatus* Andrews, 1810 is native to Africa and it is one of the most cited species in ethnobotanical surveys in Brazil (Carriconde et al. 1996). *Plectranthus neochilus* Schlecher, 1896 is also native of Africa, later introduced in Brazil (Lorenzi and Matos 2002). *Plectranthus barbatus* and *Plectranthus grandis* (Cramer, 1979) Willemse, 1985 are very similar species and they are usually confused, since they are used for the same medicinal purposes. Pioneering cytogenetic works in this genus have revealed species with diversified chromosome numbers, from 2n=14 to 84, with 2n=28 being most frequent (Morton 1962).

Cytogenetic differences among plants of the same species may reflect in variation in amount, quality and type of secondary metabolites produced by the plant, as observed by Pierre et al. (2011) in plants of *Lippia* Linnaeus, 1753.

Knowledge of DNA content, along with cytogenetics and molecular genetics, contributes to the genetic characterization of related species. The correct definition of the taxon, associated to biochemical and pharmacological evaluations, is essential for the correct use of plants for medicinal purposes. This preoccupation becomes even more important considering the recognition by the World Health Organization that about 80% of the population in developing countries makes use of plants or preparations thereof as home and communitarian remedies (Brasil 2006).

Considering the variety of chromosome number descriptions and the lack of karyotypic information about *Plectranthus* species, an enhanced investigation of the chromosome complement is necessary with the purpose of supporting taxonomic studies and evolutionary inferences. In this sense, the present work aimed at the characterization and comparison of the karyotype and DNA content of plants, from distinct localities, of the species *Plectranthus amboinicus*, *Plectranthus barbatus*, *Plectranthus grandis* and *Plectranthus neochilus*.

## Material and methods

Plants of *Plectranthus amboinicus*, *Plectranthus barbatus*, *Plectranthus grandis* and *Plectranthus neochilus* from Lavras-MG, Campinas-SP and Santa Maria-RS was cytogenetically compared. In each region three cuttings of a plant of each species were collected from plant clumps. Plants from Minas Gerais State were supplied by Medicinal Plant Garden of the University of Lavras (UFLA), the ones from Rio Grande do Sul State was provided by Medicinal Plant Garden of University of Santa Maria (UFSM) and plants from São Paulo State by the Campinas Agronomy Institute (IAC). Voucher specimens were deposited at the Research Center for Chemistry, Biology and Agriculture (CPQBA), State University of Campinas, and the State University of Campinas Herbarium (UEC), São Paulo, Brazil (Table 1). The cuttings were transplanted into vases and kept in a greenhouse. After root development, the root tips were collected and pre-treated with solution of 3 mM 8-hydroxyquinoline for 4 hours, at 4 °C, and fixed in Carnoy’s solution (3:1 /ethanol:acetic acid). The material was then stored at -20 °C for at least 24 hours.

**Table 1.** Voucher specimens numbers of *Plectranthus* plants collected.

Species	Voucher specimens
*Plectranthus amboinicus*	CPQBA 364
*Plectranthus barbatus*	UEC 121.403
*Plectranthus grandis*	CPQBA 1433
*Plectranthus neochilus*	CPQBA 1388

Slides were prepared by the squash technique, and chromosomes were stained with 1% acetic orcein after enzymatic maceration in pectinase-cellulase solution (100U:200U) for 15 min, at 37 °C.

Metaphases were digitized by means of a bright field microscope (Leica DMLS) equipped with microcamera (Nikon Digital Sight DS-Fil). The chromosomes were measured using the software Image Tool 3.0 from the UTHSCA (University of Texas Health Science Center in San Antonio).

For assembly of the karyograms and idiograms, at least four mitotic metaphases of each plant collected were used. Measurements of short and long arm length (SA/LA) were carried out for each chromosome pair, as well as of total length for each chromosome (TLi = LA + SA), total length of haploid lot (TLHL = STL_i_), and relative length (RL = TL_i_/TLHL × 100).

The data on relative length of chromosome pairs of *Plectranthus grandis* as well as of *Plectranthus barbatus* plants were compared by the least significant difference (LSD) at 5% probability, using the statistical program SAS.

Morphological classification of the chromosomes was based on centromere position, as proposed by Levan et al. (1964). Karyotypic asymmetry was evaluated according to criteria by Zarco (1986) using A_1,_ (intrachromosomal asymmetry) and A_2,_ (interchromosomal asymmetry) indices. These indices were compared to the asymmetry index (AI) proposed by Paszko (2006). Karyotypic categories were determined using the methods proposed by Stebbins (1958).

Estimation of nuclear DNA content by flow cytometry was obtained from leaf tissue according to the work of Doležel and Bartoš (2005). Nine samples of plants from each location were evaluated, total of 27 samples per specie. A total of nine samples of *Plectranthus grandis* was evaluated. Approximately 20-30 mg of young leaves of the *Plectranthus* species were used per sample, along with the same amount of *Pisum sativum* Linnaeus, 1753 leaves (internal reference standard, 2C=9.09 pg) (Doležel et al. 1992). The nuclei were released by dissociation of the plant material in Petri dish containing 1 mL of Marie and Brown (1993) cold buffer for yielding the nuclei suspension, to which were added 5 µL of RNase Type I. The suspension was stained with 25 µl of propidium iodide (1 mg/mL). For each sample, at least 10,000 nuclei were analyzed with a flow cytometer FACSCalibur (Becton Dickinson). The histograms with coefficients of variation below 0.8% were obtained using the software Cell Quest (Becton, Dickinson and Company, San Jose, CA, USA), and analyzed with the software WinMDI 2.8 (2009). The absolute DNA amount of the samples was calculated based on the values of the G1 peak means (Sample 2C DNA content = [(sample G1 peak mean)/(standard G1peak mean)] × standard 2C DNA content (pg DNA).

The data on DNA content of plants of each location, as well as the means of each species, were submitted to analysis of variance, and the mean values were compared with help of the statistical program SAS, using Tukey test at 5% probability to compare the plants and species.

## Results

**Figure 1.**
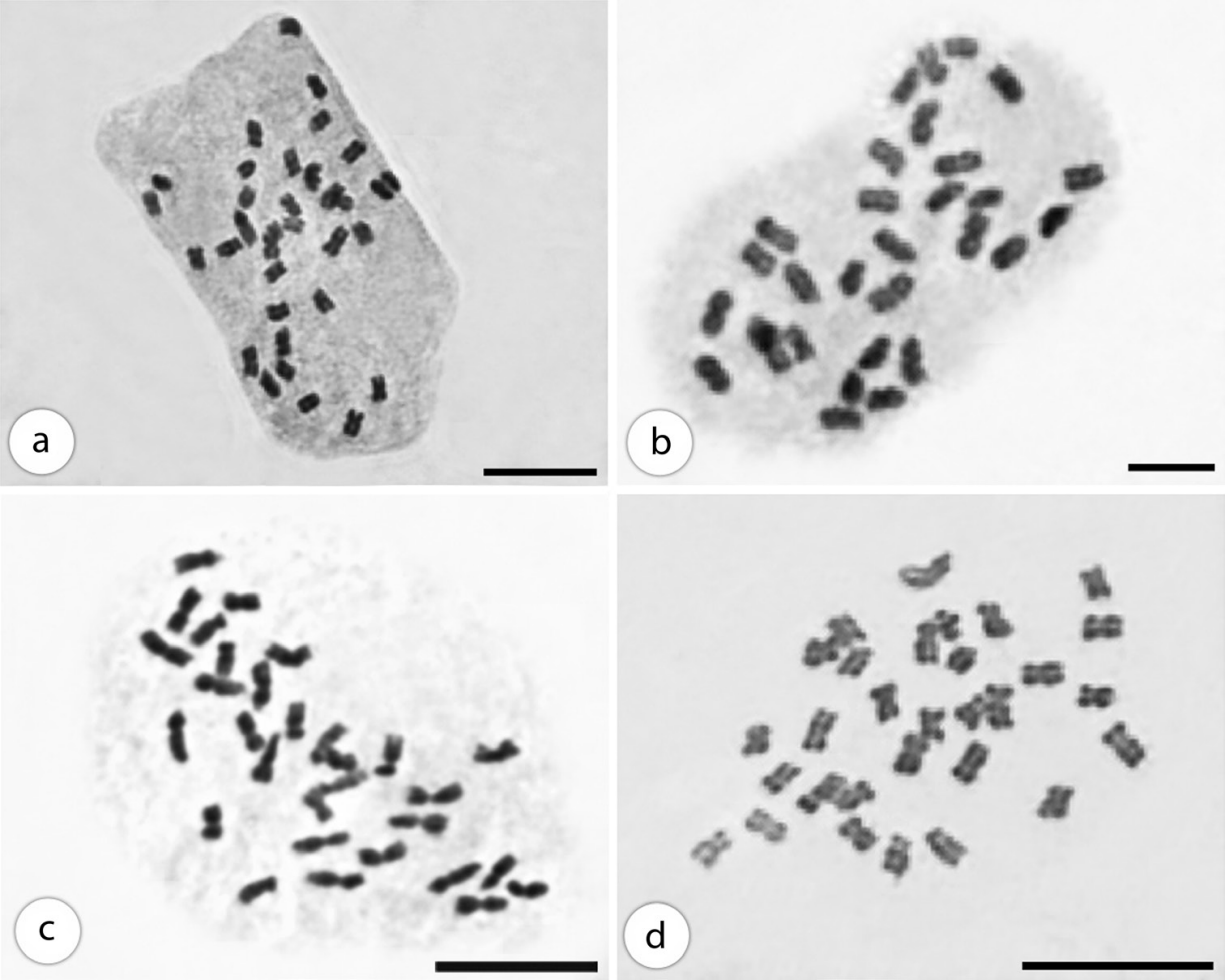
Mitotic metaphases. *Plectranthus amboinicus*, 2n=34 (**A**), *Plectranthus barbatus* (from Lavras-MG), 2n=30 (**B**), *Plectranthus grandis*, 2n=30 (**C**), *Plectranthus neochilus*, 2n=30 (**D**). Scale bars: 10 µm.

No variation was found as to the number of chromosomes among the plants of *Plectranthus* species. The somatic number of chromosomes was common (2n=30), except for *Plectranthus amboinicus*, which presented 2n=34 chromosomes (Fig. 1).

**Figure 2.**
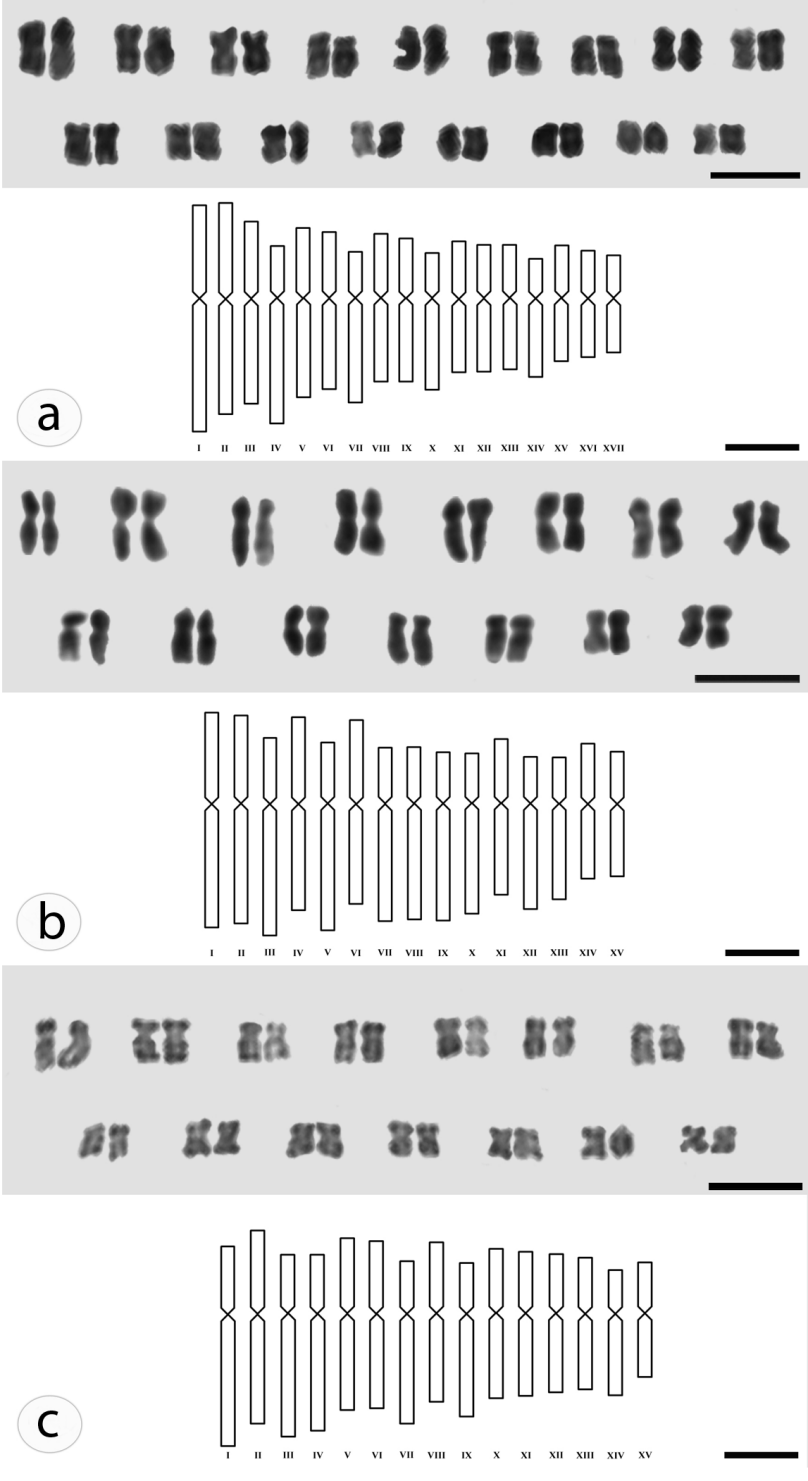
Karyograms and idiograms. *Plectranthus amboinicus* **(A)**, *Plectranthus grandis* **(B)**, *Plectranthus neochilus* **(C)**. Scale bars: Karyograms (5 µm); idiograms (1 µm).

**Figure 3.**
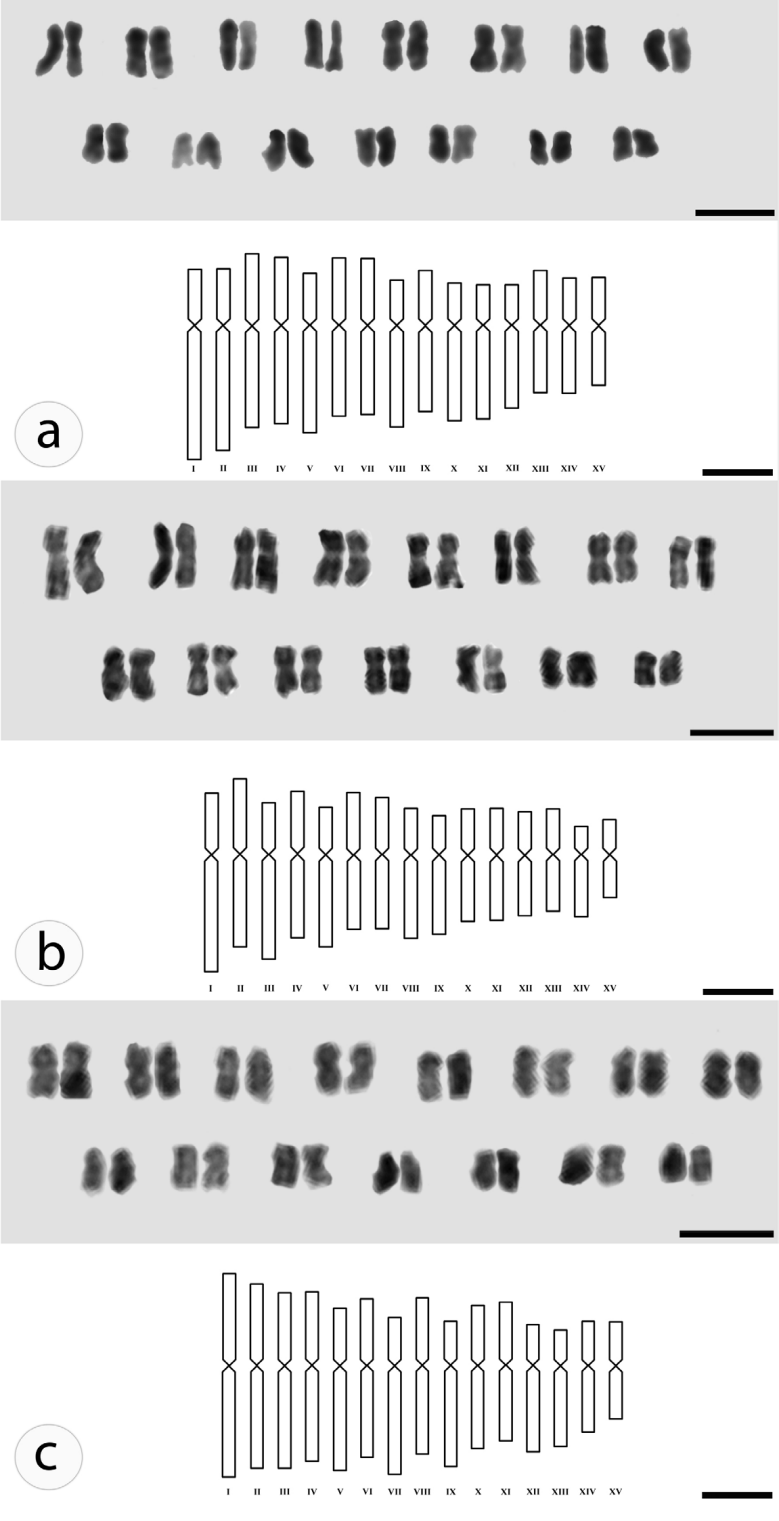
Karyograms and idiograms for *Plectranthus barbatus* from different localities. Lavras **(A)**, Campinas **(B)**, Santa Maria **(C)**. Scale bars: Karyograms (5 µm); idiograms (1 µm).

Differences were observed in chromosome morphology among the karyotypes of the species. *Plectranthus amboinicus* follows the karyotypic formula 13m+4sm, *Plectranthus grandis* 7m+8sm, and *Plectranthus neochilus* 9m+6sm (Table 2, Fig. 2). *Plectranthus barbatus* presented intraspecific variation: 8m+7sm, 9m+6sm and 10m+5sm, respectively corresponding to the specimens originated from the regions of Lavras/MG, Campinas/SP and Santa Maria/RS (Table 2, Fig. 3).

**Table 2.** Data regarding arm relation and chromosome type in species of *Plectranthus* genus.

	*P. a*	*P. b*						*P. g*		*P. n*	
		UFLA		IAC		UFSM					
1	1.43m	2.35sm		1.88sm		1.21m		1.34m		1.79sm	
2	1.21m	2.15sm		1.18m		1.25m		1.34m		1.17m	
3	1.37m	1.42m		1.98sm		1.38m		1.96sm		2.00sm	
4	2.35sm	1.44m		1.29m		1.28m		1.20m		2.13sm	
5	1.39m	2.07sm		1.88sm		1.79sm		2.02sm		1.13m	
6	1.36m	1.32m		1.17m		1.37m		1.18m		1.19m	
7	2.19sm	1.32m		1.30m		2.23sm		2.07sm		1.92sm	
8	1.27m	2.22sm		1.79sm		1.30m		1.98sm		1.11m	
9	1.38m	1.53m		1.97sm		2.22sm		2.27sm		1.81sm	
10	1.99sm	2.19sm		1.40m		1.35m		2.15sm		1.25m	
11	1.29m	2.25sm		1.38m		1.17m		1.37m		1.26m	
12	1.36m	2.01sm		1.41m		2.06sm		2.20sm		1.39m	
13	1.29m	1.20m		1.21m		2.27sm		2.04sm		1.43m	
14	1.97sm	1.43m		2.11sm		1.47m		1.21m		1.84sm	
15	1.17m	1.24m		1.20m		1.21m		1.35m		1.25m	
16	1.18m										
17	1.24m										

* *P. a* (*Plectranthus amboinicus*); *P. b* (*Plectranthus barbatus*); *P. g* (*Plectranthus grandis*); *P. n* (*Plectranthus neochilus*); m = metacentric ; sm = submetacentric .

The position of the centromere was coincident among the four species only for the pairs 6 and 15, with these being classified as metacentric (Table 2).

Also among the plants of *Plectranthus barbatus* little karyotypic similarity could be established. Only the pairs 4, 5, 6 and 15 had chromosomes with coinciding classification. Moreover, these same pairs are also coincident in *Plectranthus grandis*, which presented greater karyotypic similarity with the plants of *Plectranthus barbatus* (Santa Maria), differing only in the pairs 3, 8 and 10 (Table 2).

The contrasts accomplished through the statistical test of least significant difference (LSD) among the plants revealed that *Plectranthus barbatus* (Campinas) differs statistically from *Plectranthus grandis* in relation to the pairs 2 and 12. The pair 2 presents relative length with mean values of 8.78 and 8.10, and the pair 12 shows 5.45 and 5.92 for *Plectranthus barbatus* and *Plectranthus grandis*, respectively. The pair 8 differed among the plants of *Plectranthus barbatus* originated from Lavras and Santa Maria, with respective averages of 6.68 and 6.93 (Tables 3–4). The remaining pairs did not present significant differences for this variable among the plants of the species.

**Table 3.** Data regarding the total length (µm) and relative length (%) of each chromosome of *Plectranthus* spp.

Pair	*Plectranthus amboinicus*		*Plectranthus barbatus*						*Plectranthus grandis*		*Plectranthus neochilus*	
			UFLA		IAC		UFSM					
1	3.04	(8.89)	2.99	(8.56)	3.57	(9.42)	2.93	(9.09)	2.95	(8.37)	2.82	(8.55)
2	2.87	(8.38)	2.87	(8.22)	3.33	(8.78)	2.63	(8.15)	2.85	(8.10)	2.75	(8.33)
3	2.49	(7.26)	2.75	(7.87)	3.09	(8.15)	2.52	(7.82)	2.72	(7.72)	2.55	(7.75)
4	2.42	(7.06)	2.60	(7.44)	2.94	(7.75)	2.43	(7.55)	2.65	(7.52)	2.42	(7.34)
5	2.31	(6.73)	2.52	(7.20)	2.80	(7.38)	2.34	(7.26)	2.58	(7.32)	2.41	(7.31)
6	2.13	(6.23)	2.51	(7.17)	2.72	(7.17)	2.27	(7.03)	2.50	(7.09)	2.36	(7.15)
7	2.05	(5.98)	2.44	(6.97)	2.61	(6.89)	2.26	(7.00)	2.37	(6.72)	2.30	(6.97)
8	2.02	(5.90)	2.34	(6.68)	2.59	(6.83)	2.23	(6.93)	2.36	(6.69)	2.25	(6.82)
9	1.94	(5.68)	2.23	(6.37)	2.35	(6.20)	2.10	(6.50)	2.31	(6.57)	2.14	(6.48)
10	1.86	(5.42)	2.18	(6.23)	2.25	(5.95)	2.06	(6.39)	2.18	(6.19)	2.04	(6.19)
11	1.78	(5.20)	2.11	(6.02)	2.23	(5.87)	2.00	(6.21)	2.14	(6.09)	1.97	(5.97)
12	1.72	(5.02)	1.95	(5.58)	2.07	(5.45)	1.84	(5.71)	2.09	(5.92)	1.89	(5.72)
13	1.68	(4.90)	1.94	(5.56)	2.03	(5.35)	1.66	(5.15)	1.95	(5.52)	1.78	(5.39)
14	1.61	(4.70)	1.83	(5.23)	1.78	(4.71)	1.57	(4.88)	1.86	(5.29)	1.74	(5.26)
15	1.57	(4.59)	1.71	(4.89)	1.56	(4.10)	1.40	(4.34)	1.72	(4.89)	1.57	(4.76)
16	1.45	(4.22)										
17	1.32	(3.85)										

**Table 4.** Comparison of relative lengths of the chromosome pairs 2, 8 and 12 of *Plectranthus* plants by LSD test.

	*P. b* (UFLA)	*P. b* (IAC)	*P. b* (UFSM)	*P. g* (IAC)
*P. b* (UFLA)	-	ABC	AbC	ABC
*P. b* (IAC)	ABC	-	ABC	aBc
*P. b* (UFSM)	AbC	ABC	-	ABC
*P. g* (IAB)	ABC	aBc	ABC	-

* Lowercase letters indicate statistically different mean values. Pair 2 (A); Pair 8 (B); Pair 12 (C). *P. b* (*Plectranthus barbatus*); *P. g* (*Plectranthus grandis*).

According to Stebbins (1958), *Plectranthus amboinicus* and *Plectranthus barbatus* (Campinas and Santa Maria) have karyotypes included in the category 3b. Differently, the karyotypes of *Plectranthus neochilus*, *Plectranthus grandis* and *Plectranthus barbatus* (Lavras) were included in the category 3a.

The studied species of *Plectranthus* have close karyotypic asymmetry indices (Table 5).

**Table 5.** Values of karyotypic asymmetry indices of *Plectranthus* species, according to criteria proposed by Zarco (1986) (A1: intrachromosomal asymmetry, A2: interchromosomal asymmetry) and proposed by Paszko (2006) (AI: asymmetry index).

	*P. a*	*P. b*			*P. g*	*P. n*
		UFLA	IAC	UFSM		
A_1_	0.29	0.39	0.32	0.32	0.38	0.33
A_2_	0.23	0.17	0.22	0.19	0.15	0.16
AI	3.09	2.60	2.93	2.82	2.48	2.06

* *P. a* (*Plectranthus amboinicus*); *P. b* (*Plectranthus barbatus*); *P. g* (*Plectranthus grandis*); *P. n* (*Plectranthus neochilus*).

*Plectranthus amboinicus* has the largest difference in relation to total size of the chromosomes, besides having the greatest asymmetry index as proposed by Paszko (2006). *Plectranthus neochilus* has one of the smallest values, both for intrachromosomal (A_1_) and interchromosomal asymmetry (A_2_), as proposed by Zarco (1986). It also has the smallest asymmetry index (AI) value as described by Paszko (2006) (Table 5).

**Figure 4.**
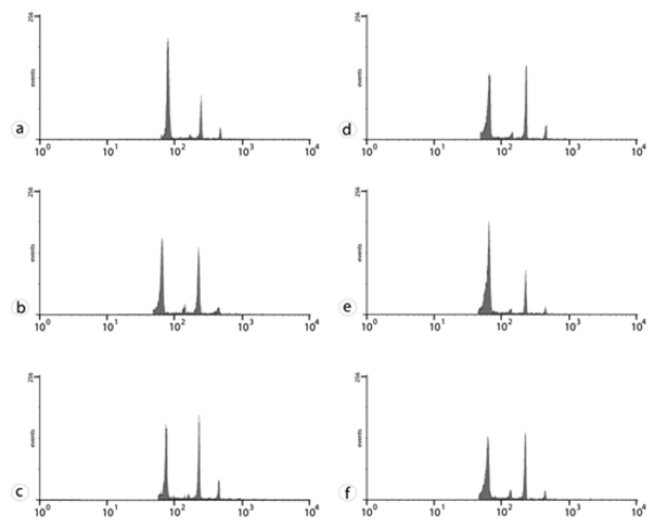
Flow cytometry histograms. *Plectranthus amboinicus* **(A)**, *Plectranthus grandis* **(B)**, *Plectranthus neochilus* **(C)**, *Plectranthus barbatus* **(D–F)** from UFLA **(D)**, IAC **(E)**, UFSM **(F)**. The first peak in each histogram refers to the G1 peak of each of the *Plectranthus* species, and the second G1 peak corresponds to the reference sample (*Pisum sativum*). The abscissa represents the DNA amount, and the ordinate the number of nuclei.

In relation to DNA amount in the evaluated *Plectranthus* plants, two groupings were identified (Table 6): *Plectranthus amboinicus* (5.86 pg) and *Plectranthus neochilus* (5.98 pg) had the highest mean values (Fig. 4a, c), whereas *Plectranthus grandis* (5.23 pg) and *Plectranthus barbatus* (5.35 pg) had the lowest (Fig. 4b, d–f).

**Table 6.** Mean values of 2C DNA and coefficient of variation obtained by flow cytometry technique for *Plectranthus* plants.

Species/Plant	DNA (pg)^1^	CV (%)
*Plectranthus amboinicus* (UFLA)	5.98 a	0.79
*Plectranthus amboinicus* (IAC 465)	5.79 a	0.72
*Plectranthus amboinicus* (IAC 2193)	5.81 a	0.57
Mean	5.86 A	
*Plectranthus barbatus* (UFLA)	5.20 a	0.57
*Plectranthus barbatus* (IAC)	5.17 a	0.70
*Plectranthus barbatus* (UFSM)	5.69 b	0.57
Mean	5.35 B	
*Plectranthus grandis*	5.23 B	0.64
*Plectranthus neochilus* (UFLA)	5.99 a	0.56
*Plectranthus neochilus* (IAC)	5.94 a	0.54
*Plectranthus neochilus* (UFSM)	6.00 a	0.55
Mean	5.98 A	

1 Averages followed by the same lowercase letters within each group of species, and averages followed by the same capital letters do not differ statistically by Tukey test at 5% probability.

## Discussion

Distinct chromosome numbers for *Plectranthus amboinicus* have already been described in the literature (2n=16, 24, 30, 32, 34, 48 and 56) in works that treated the species with different synonymies (Scheel 1931, Basavaraja and Krishnappa 1982, Saggoo and Bir 1983, Thoppil 1993).

Thoppil (1993), studying the synonymy *Coleus aromaticus* Bentham, 1831, found 2n=32 as most common chromosome number and that the relative lengths of the largest and smallest chromosomes were 8.19 and 4.37, respectively. The values of relative length described here, for plants with 34 chromosomes, are slightly similar to those found by Thoppil (1993) (Table 2), suggesting the occurrence of rearrangements of breakage or fusion type in the chromosomes of intermediary size.

Karyotypic studies on *Plectranthus neochilus* are rare in the literature. De Wet (1958) and Riley and Hoff (1961), using the synonymy *Coleus pentheri* Gürke, 1905 described 32 chromosomes for plants originated from the east and south of Africa. These authors did not report details of chromosome morphology for this species. The occurrence of 30 chromosomes in *Plectranthus neochilus* is reported for the first time in the present work.

The occurrence of 30 chromosomes in *Plectranthus barbatus* that was observed for different accessions of this species in the present study corroborates the number reported earlier by different authors (Cherian and Kuriachan 1981, Sagoo and Bir 1983, Bahl and Tyagi 1988, Thoppil 1993). Other descriptions regarding variation in chromosome number (2n=28 to 34) have been related for *Plectranthus barbatus* (Reddy 1951, Saggoo and Bir 1983). Riley and Hoff (1961) were the first to find a specimen with 32 chromosomes. According to Thoppil (1993), specimens of this species from the south of India with 28 chromosomes, have autotetraploid genome with basic number x=7.

The statistical differences observed for the pairs 2, 8 and 12 in *Plectranthus barbatus* and *Plectranthus grandis* suggest the occurrence of chromosomal rearrangements, seeing that some of these pairs present variation both in relative length as well as in centromeric position. This way, the chromosomes of pairs 8 and 12 classified as submetacentric may have undergone alterations, namely deficiency in one of the chromosome arms, giving rise to the metacentric form, or duplications in one of the arms of these chromosomes, thus rendering them submetacentric. The differences seen in the pair 2 for *Plectranthus barbatus* (Campinas) and *Plectranthus grandis* did not express variations in centromere position, which suggests events of duplication/deficiency in both chromosome arms.

The remaining chromosome pairs of the evaluated *Plectranthus barbatus* and *Plectranthus grandis* plants did not present significant statistical differences regarding relative length. Nevertheless, the centromere position in some of the pairs of *Plectranthus barbatus* plants and of the *Plectranthus grandis* plant appeared altered. Taking the chromosome pair 1 as example, the plants from Lavras and Campinas had it classified as submetacentric, and that from Santa Maria, together with *Plectranthus grandis*, had the same pair classified as metacentric. These changes in classification of the chromosome pair as to centromere position may be justified by the occurrence of inversions, since the relative lengths are statistically similar. Also, other mechanisms may drastically modify the chromosome structure, among which centromeric repositioning, as reported by Rocchi et al. (2012). These authors propose that centromeric repositioning is an alteration that occurs in the chromosome structure without changes in the base sequence of the DNA. This event creates a new centromere (neocentromere), apparently through epigenetic factors, and substitutes the original one. This finding has deeply modified the interpretation of karyotypic evolution in various mammals.

Different pressures exerted by the different environments can be other reason for karyotypic variations mentioned for *Plectranthus barbatus*, since this hypothesis was considered previously by Shah (1989), who evaluated populations of this species from distinct geographical origins and identified variation in the chromosome morphology among plants of this species.

Passinho et al. (1999) and Bandeira et al. (2010) also evaluated different populations of *Plectranthus* species by means of AFLP (Amplified Fragment Length Polymorphism) and RAPD (Random Amplified Polymorphic DNA), respectively, and according to these authors there are intra and interpopulational genetic variation.

The occurrence of differentiated karyotypic formulas for plants of *Plectranthus barbatus* and the fact that *Plectranthus barbatus* presents nuclear DNA content statistically similar to that of *Plectranthus grandis*, are not able to indicate that *Plectranthus barbatus* and *Plectranthus grandis* have enough differences to be considered distinct species. Therefore, more experiments using molecular cytogenetic techniques are needed in order to understand the relationship between both species.

Regarding to asymmetry of karyotype, based on the methods of Stebbins (1958), *Plectranthus amboinicus* is included in a more asymmetric category in relation to the other studied species. According to Stebbins (1958), the karyotypic symmetry is characterized by the predominance of metacentric and submetacentric chromosomes of approximately same size. Nonetheless, this species presents the largest proportion between the smallest and largest chromosome, thus being interchromosomally more asymmetrical.

Based on asymmetry indexes proposed by Zarco (1986) and Paszko (2006), *Plectranthus amboinicus* also exhibited the most asymmetric karyotype, as it presented the highest AI value (Table 5). According to Paszko (2006), high values are indicative of more elevated levels of karyotypic heterogeneity. *Plectranthus neochilus* can be considered the species with lowest asymmetry in relation to the others, both from the intrachromosomal as well as interchromosomal point of view, due to presenting the lowest coefficients of variation for centromeric index and total chromosome length; these data consequently generate a value inferior to AI (Table 5). Both species are the most distant ones in terms of karyotypic asymmetry. These species have probably undergone structural rearrangements of karyotype, without great losses or gains of DNA sequences, as both have statistically similar amounts of nuclear DNA. Even though the dispersion diagram may indicate different degrees of asymmetry, the studied *Plectranthus* species are strictly related, which can be observed by the gradual variations in AI values (Table 5).

The variation among karyotypes of kindred species and among plants, associated with the differences in nuclear DNA content found in this work, supports the hypothesis that, karyotypically, *Plectranthus amboinicus* and *Plectranthus neochilus* are more stable species and the variation found among plants of *Plectranthus barbatus*, regarding chromosome morphology, express differences among populations.

## Conclusions

The populations of *Plectranthus amboinicus* and *Plectranthus neochilus* present coinciding karyotypes among their respective plants.

*Plectranthus barbatus* is a species undergoing active process of karyotypic variation.

The karyotypic intraspecific variation in *Plectranthus barbatus* is an indication that *Plectranthus grandis* is one of the events of variation in the species, since the species exhibit the same morphological characteristics.
